# Analysis and Identification of Active Compounds from Gami-Soyosan Toxic to MCF-7 Human Breast Adenocarcinoma Cells

**DOI:** 10.3390/biom9070272

**Published:** 2019-07-10

**Authors:** Mi-Yeon Jung, Chang-Seob Seo, Seon-Eun Baek, Jaemin Lee, Myoung-Sook Shin, Ki Sung Kang, Sullim Lee, Jeong-Eun Yoo

**Affiliations:** 1 Department of Obstetrics and Gynecology, College of Korean Medicine, Daejeon University, Daejeon 35235, Korea; 2 Herbal Medicine Research Division, Korea Institute of Oriental Medicine, Daejeon 34054, Korea; 3 College of Korean Medicine, Gachon University, Seongnam 13120, Korea; 4 Department of Life Science, College of Bio-Nano Technology, Gachon University, Seongnam 13120, Korea

**Keywords:** Gami-soyosan, gallic acid, decursin, decursinol angelate, breast cancer, MCF-7, apoptosis

## Abstract

Gami-soyosan is a medicinal herbal formulation prescribed for the treatment of menopausal symptoms, including hot flashes and osteoporosis. Gami-soyosan is also used to treat similar symptoms experienced by patients with breast cancer. The incidence of breast cancer in women receiving hormone replacement therapy is a big burden. However, little is known about the components and their mechanism of action that exhibit these beneficial effects of Gami-soyosan. The aim of this study was to simultaneously analyze compounds of Gami-soyosan, and determine their cytotoxic effects on estrogen receptor (ER)-positive MCF-7 human breast adenocarcinoma cells. We established a simultaneous analysis method of 18 compounds contained in Gami-soyosan and found that, among the various compounds in Gami-soyosan, gallic acid (**1**), decursin (**17**), and decursinol angelate (**18**) suppressed the viability of MCF-7 cells. Gallic acid (**1**), decursin (**17**), and decursinol angelate (**18**) induced apoptotic cell death and significantly increased poly (ADP-ribose) polymerase (PARP) cleavage and the Bcl-2-associated X protein/ B-cell lymphoma 2 (Bax/Bcl-2) ratio. Decursin (**17**) increased the expression of cleaved caspases-8, -9, -7, and -3. Decursinol angelate (**18**) increased the expression of cleaved caspase-8 and -7. These three components altered the different apoptosis signal pathways. Collectively, gallic acid (**1**), decursin (**17**), and decursinol angelate (**18**) may be used to inhibit cell proliferation synergistically in patients with ER-positive breast cancer.

## 1. Introduction

Breast cancer originates in breast ducts and lobes, and is a very common cancer that accounts for 25.2% of all gynecological cancers worldwide. It is generally known that factors, such as older age, history of contralateral breast cancer, late menopause, family history, and hormone replacement therapy (HRT), increase the risk of breast cancer. Before menopause, the risk of breast cancer incidence increases two-fold every decade [1,2,3].

Among the types of breast cancer, estrogen receptor (ER)-positive breast cancer accounts for more than a 70% incidence rate. Moreover, estrogen plays a significant role in the growth and development of cancer cells [4]. Tamoxifen, which inhibits the action of estrogen, and aromatase inhibitors, which block estrogen production, are usually used to treat breast cancer via endocrine therapies [5].

However, they may result in menopausal symptoms, including facial flushing, vaginal dryness, insomnia, and osteoporosis, which can lead to a lower quality of life and depression in patients with breast cancer [6]. Although HRT has been reported to increase the risk of breast cancer [7], it is utilized to treat menopausal symptoms, such as osteoporosis and hot flashes [8,9]. Therefore, research into therapeutic agents that can ameliorate menopausal symptoms and decrease the risk of breast cancer is currently required.

According to the “Bangyakhappyeon”, the Korean traditional medicine book published in 19th century, Gami-soyosan has been shown to be an effective treatment of various menopausal symptoms [10,11]. Hence, Gami-soyosan has been extensively used in traditional Korean medicine to improve menopausal symptoms, including hot flashes, sleep disturbances, headache, and dizziness, in postmenopausal women [10,11,12]. Previous studies have shown that the formula has preventative and ameliorative effects on osteoporosis, neuroprotection, and anti-stress and antipyretic actions [13,14]. In addition, Chen suggested that Jia-Wei-Xiao-Yao-San (JWXYS), the composition of which is similar to that of Gami-soyosan, may affect MCF-7 cell proliferation [15].

Recently, Gami-soyosan has also been applied in practice for the treatment of menopause-like symptoms in patients with breast cancer. In this case, Gami-soyosan improves the symptoms without exacerbation of cancer in breast cancer patients [16,17,18]. However, to our knowledge, no study has reported the cytotoxic effect of the compounds of Gami-soyosan in breast cancer. Therefore, we evaluated the novel actions of Gami-soyosan and assessed its potential utilization in assisting in the improvement of symptoms associated with ER-positive breast cancer by inhibiting proliferation of MCF-7 breast cancer cells, which is the most commonly used ER-positive breast cancer cell line [19]. Therefore, in this study, we performed a simultaneous analysis of Gami-soyosan components and determined their toxicity against MCF-7 human breast adenocarcinoma cells.

## 2. Materials and Methods

### 2.1. Gami-Soyosan Sample

The Gami-soyosan decoction was prepared in the Korea Institute of Oriental Medicine (KIOM), as described in our previous study [20]. Briefly, the 12 raw herbs of Gami-soyosan (Table 1) were obtained from Kwangmyungdang Medicinal Herbs (Ulsan, Korea) and identified by Professor Jung-Hoon Kim of the School of Korean Medicine, Pusan National University (Yangsan, Korea). A voucher specimen of each raw material (from 2012–KE45–1 to KE45–12) and the Gami-soyosan decoction (2012–KE45) were stored at the KIOM. To obtain the Gami-soyosan water decoction, the mixture of 12 herbs was extracted in distilled water at 100 °C for 2 h under pressure (98 kPa) using an electric extractor (COSMOS-660; Kyungseo Machine Co., Incheon, Korea). The extract solution was freeze-dried by using a LP100R freeze dryer (IlShinBioBase, Yangju, Korea) to afford 970.4 g of the Gami-soyosan extract.

### 2.2. Chemicals

**Figure 1:**
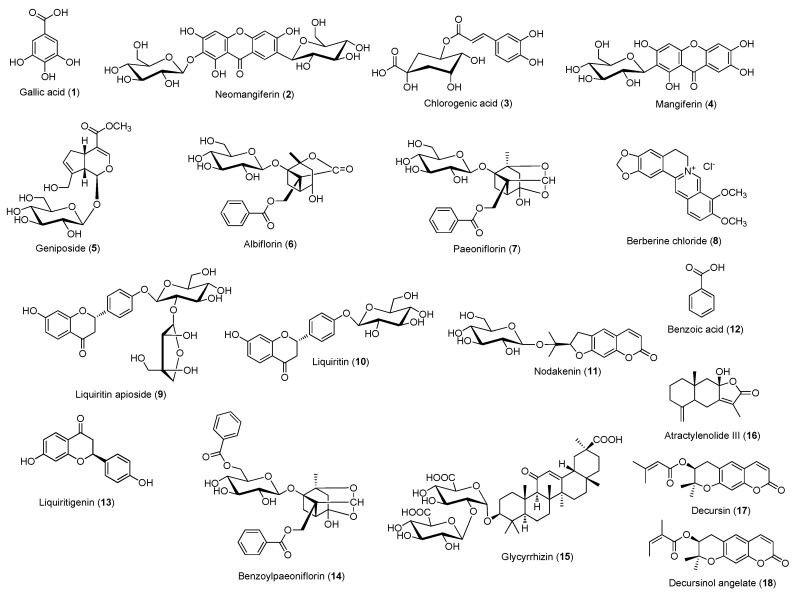
Chemical structures of compounds **1**–**18** present in Gami-soyosan.

Gallic acid (**1**), berberine chloride (**8**), and benzoic acid (**12**) were obtained from Sigma-Aldrich (St Louis, MO, USA). Neomangiferin (**2**), mangiferin (**4**), liquiritigenin (**13**), and benzoylpaeoniflorin (**14**) were obtained from Chengdu Biopurify Phytochemicals (Chengdu, China). Geniposide (**5**), albiflorin (**6**), paeoniflorin (**7**), liquiritin (**10**), and glycyrrhizin (**15**) were obtained from Wako (Osaka, Japan). Liquiritin apioside (**9**) and atractylenolide III (**16**) were obtained from Shanghai Sunny Biotech (Shanghai, China). Chlorogenic acid (**3**, 99.6% purity) was obtained from Acros Organics (Pittsburgh, PA, USA), and nodakenin (**11**, 98.0% purity), decursin (**17**), and decursinol angelate (**18**) were obtained from NPC Bio-Technology (Yeongi, Korea). The chemical structures of each compound are shown in Figure 1, and the purity of these compounds was higher than 98%. The distilled water, acetonitrile, and methanol used for the mobile phase and formic acid were purchased from J.T. Baker (Phillipsburg, NJ, USA) and Merck KGaA (Darmastadt, Germany), respectively.

### 2.3. HPLC Analysis of Gami-Soyosan

High-performance liquid chromatography (HPLC) analysis for 18 marker components for quality assessment of the Gami-soyosan sample are described in detail in our previous study [20]. In brief, simultaneous determination was carried out using an LC-20A Prominence HPLC system (Shimadzu Corp., Kyoto, Japan) equipped with a photo diode array (PDA) detector and the analytical parameters for simultaneous analysis are shown in Table 2.

### 2.4. Cells and Cell Culture

MCF-7 cells are from a human breast adenocarcinoma cell line obtained from the American Type Culture Collection (ATCC, Bethesda, MD, USA). They were cultured in Roswell Park Memorial Institute 1640 medium (RPMI 1640; Corning, Manassas, VA, USA) containing 10% fetal bovine serum (Gibco BRL, Carlsbad, MD, USA) and 1% penicillin/streptomycin solution (1000 IU/mL penicillin and 10,000 μg/mL streptomycin; Life Technologies, Waltham, MA, USA) in a CO_2_ incubator at 37 ℃. In all cell experiments, dimethyl sulfoxide (DMSO) was used as a vehicle to dissolve samples. The final concentration of DMSO was kept under 0.1%, which exerts no effect of the vehicle (DMSO) compared to the naive cell.

### 2.5. Determination of Cell Viability

The Ez-Cytox assay kit, which is a WST-based Cell Viability/Cytotoxicity Assay Kit (Daeil Lab Service Co., Seoul, Korea), was used for quantifying cell viability. MCF-7 cells (1 × 10^4^ cells/well) were seeded into a 96-well plate and cultured until confluent. Vehicle (dimethyl sulfoxide, DMSO) or indicated concentrations of each compound were added into the wells and incubated for 24 h. Subsequently, Ez-Cytox solution was added into each well and the cells were incubated for 1 h. The absorbance at 450 nm compared with absorbance of a reference at 600 nm was determined through a SPARK 10 M microplate reader (Tecan Group Ltd., Männedorf, Switzerland). Cell viability was calculated based on a ratio to 100% of the vehicle control (DMSO).

### 2.6. Image-Based Cytometric Assay

MCF-7 cells (1 × 10^6^ cells/well) were seeded into a 6-well plate and cultured for 24 h. Vehicle (DMSO) or indicated concentrations of each compound were added into the wells and incubated for 24 h. Following incubation, the cells were detached and then stained with an image-based cytometric assay kit (Invitrogen, Temecula, CA, USA) in the dark for 20 min at 20 ± 5 °C. The presence of apoptotic cells was determined using TaliImage-based cytometer (Invitrogen, Carlsbad, CA, USA) and analyzed with TaliPCApp (version 1.0, Invitrogen, Carlsbad, CA, USA). The ratio of apoptotic cells was calculated based on a ratio to 100% of the vehicle control (DMSO).

### 2.7. Western Blotting

MCF-7 cells (1 × 10^6^ cells/well) were seeded into a 6-well plate and cultured for 24 h. Vehicle (DMSO) or indicated concentrations of each compound were added into the wells and incubated for 24 h. Following incubation, the cells were collected and lysed with RIPA buffer (Elpis Biotech, Daejeon, Korea). The protein concentrations of lysates were determined using a Pierce™ BCA protein assay kit (Thermo Scientific, Carlsbad, CA, USA). Equal amounts of protein and sample buffer (4× NuPAGE LDS, Thermo Scientific) were mixed and boiled for 5 min at 95 °C. The samples were separated by sodium dodecyl sulfate-polyacrylamide gel electrophoresis using acrylamide gels and transferred to polyvinylidene fluoride membranes (Merck Millipore, Darmstadt, Germany). After blocking with 2.5% skim milk, membranes were blotted with specific primary antibodies antibody against poly (ADP-ribose) polymerase (PARP), Bcl-2-associated X protein (Bax), B-cell lymphoma 2 (Bcl-2), cleaved caspase-3, -7, -8, -9, and β-actin (Cell Signaling Technology, Inc., Danvers, MA, USA) for 6 h at 20 ± 5 ℃. After washing, the membranes were incubated with the horseradish peroxidase-conjugated secondary antibody (Cell Signaling Technology, Inc.) at 20 ± 5 ℃ for 1 h. The blotted membrane was developed with Pierce ECL Western Blotting Substrate (Rockford, IL, USA) and visualized with the chemiluminescence System (FUSION Solo; PEQLAB Biotechnologie GmbH, Erlangen, Germany).

### 2.8. Statistical Analysis

All quantitative data are presented as the mean ± standard deviation (S.D.) (*n* = 3). Comparisons between the groups were performed using repeated measures analysis of variance (ANOVA). When a significant interaction was present, Dunnett’s post-hoc test was performed. The test was considered statistically significant when *p* < 0.05 as compared to untreated cells.

## 3. Results and Discussion

Breast cancer can be categorized as ER-positive or ER-negative based on the presence or absence of ER expression, respectively; usually, breast cancer is ER-positive [21]. The development of ER-positive breast cancer is closely related to the action of estrogen. High estrogen levels in the blood or excessive estrogen exposure leads to an increased risk of developing breast cancer [22]. Estrogen directly or indirectly increases the risk of genetic mutations associated with cell proliferation and apoptosis in the breast tissue. In addition, estrogen induces cell proliferation through the stimulation of growth factors in breast cancer cells and the suppression of TNF-α, which induces apoptosis. Estrogen facilitates cancer cell survival by conferring insensitivity to cell death signaling [23].

In addition, estrogen is widely used as the most effective treatment for menopausal symptoms [9]; consequently, side effects have been reported. Estrogen-progestin therapy, which is a type of HRT, is known to increase the risk of invasive breast cancer [7,8]. Studies have also reported that HRT increases the risk of breast cancer [8,9].

Generally, therapeutic agents that suppress the action and production of estrogen are utilized to treat patients with ER-positive breast cancer [24]. Tamoxifen is a representative drug that works as an ER antagonist in the prevention and therapy of breast cancer. This therapy has contributed significantly to the reduction in breast cancer recurrence and mortality [25,26]. Aromatase inhibitors, which are agents that suppress the production of estrogen, are utilized as alternatives to tamoxifen in postmenopausal women. Aromatase converts other hormones to estrogen. In postmenopausal women, aromatase is the main regulator of estrogen production, whereas aromatase inhibitors suppress estrogen production [27,28]. However, when these drugs are used to treat breast cancer through estrogen inhibition, estrogen deficiency symptoms, such as vaginal dryness, unstable emotion, osteoporosis, and arthritis, are observed. Because these symptoms cannot be treated with HRT, as is usual in postmenopausal women, other treatments are required [29]. In traditional Korean medicine, Gami-soyosan is prescribed to ameliorate menopausal symptoms, especially for the treatment of hot flashes [30]. Gami-soyosan is used not only for menopausal women, but also for patients with breast cancer who experience symptoms similar to menopause symptoms [16,17,18].

**Figure 2:**
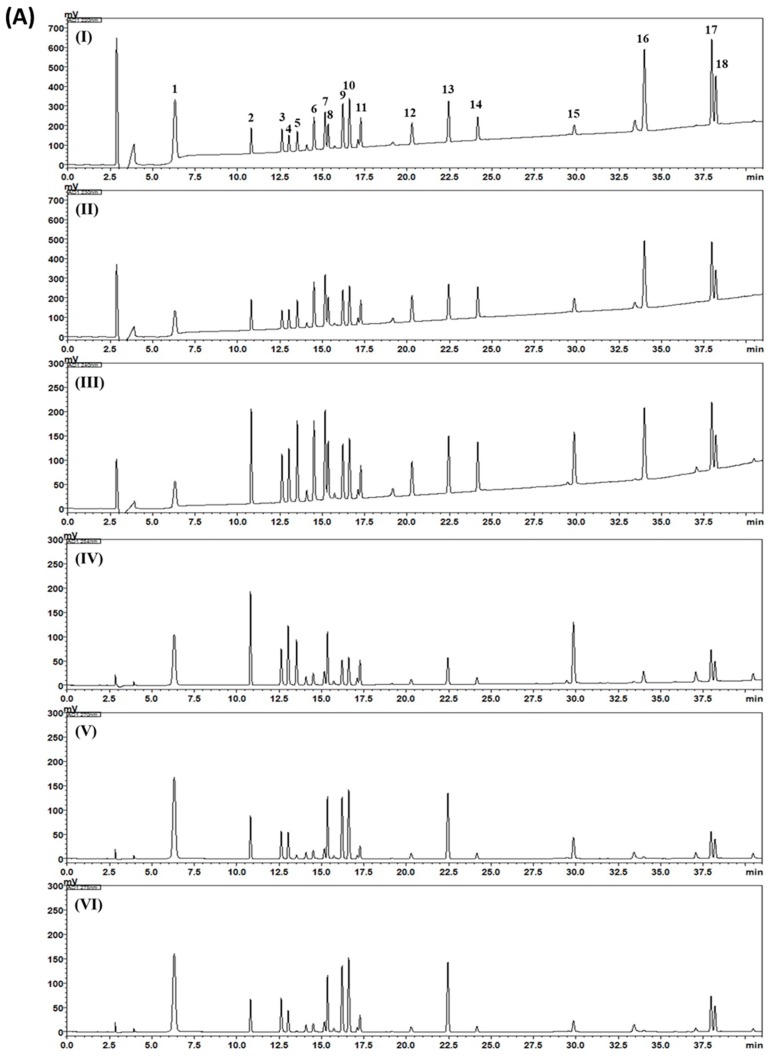

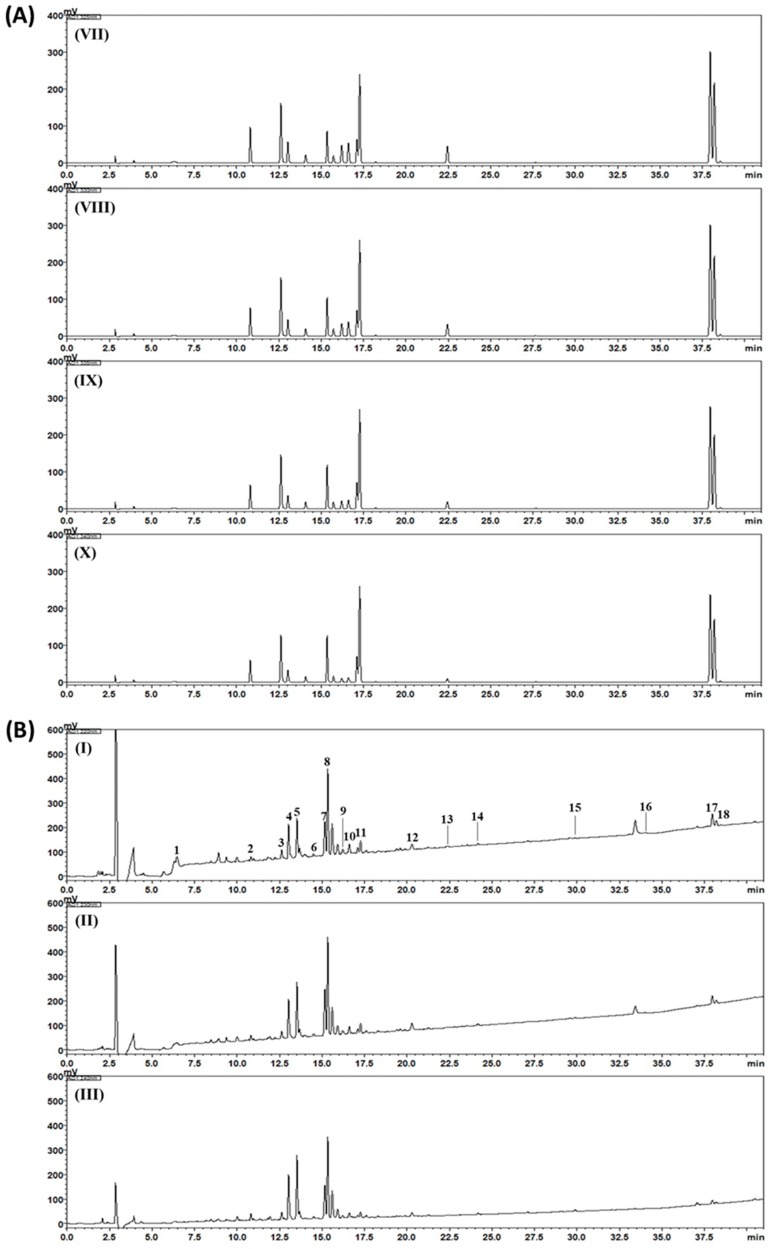

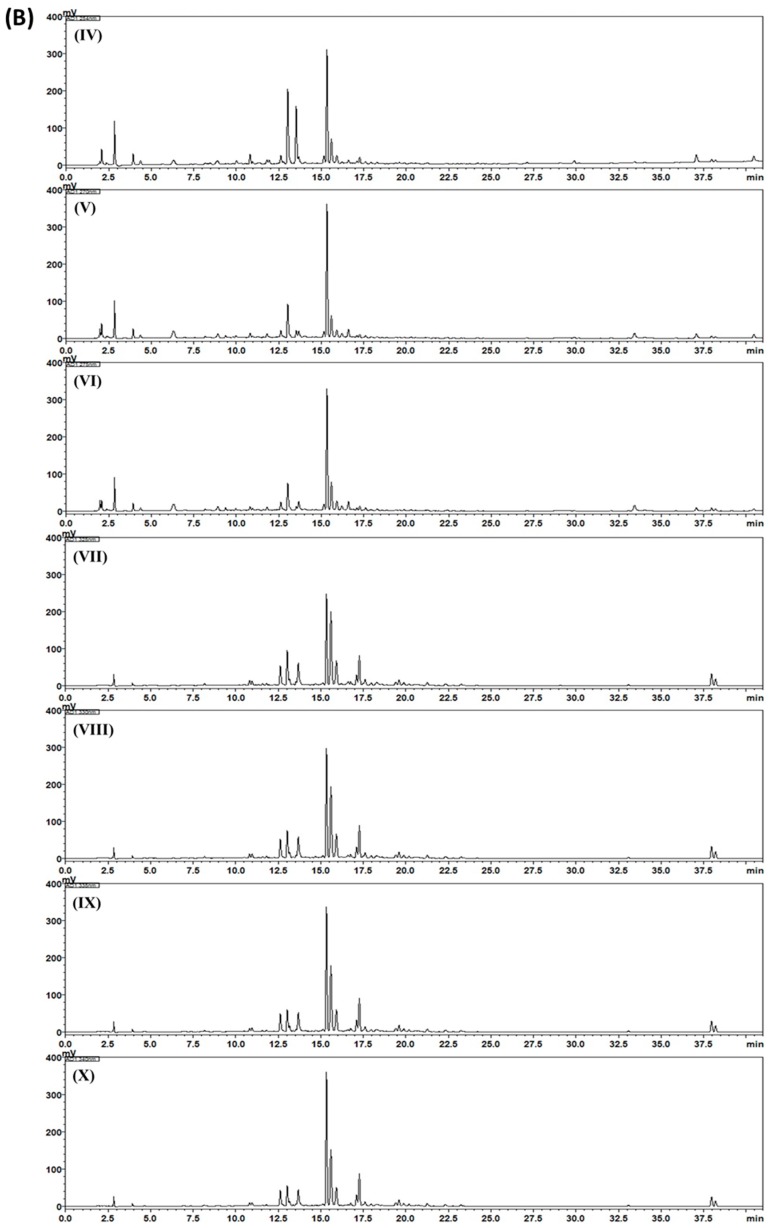
Representative high-performance liquid chromatography (HPLC) chromatograms of the standard solution (**A**) and Gami-soyosan sample (**B**) at a 220 (I), 230 (II), 240 (III), 254 (IV), 270 (V), 275 (VI), 325 (VII), 330 (VIII), 335 (IX), and 340 (X) nm. Gallic acid (**1**), neomangiferin (**2**), chlorogenic acid (**3**), mangiferin (**4**), geniposide (**5**), albiflorin (**6**), paeoniflorin (**7**), berberine chloride (**8**), liquiritin apioside (**9**), liquiritin (**10**), nodakenin (**11**), benzoic acid (**12**), liquiritigenin (**13**), benzoylpaeoniflorin (**14**), glycyrrhizin (**15**), atractylenolide III (**16**), decursin (**17**), and decursinol angelate (**18**).

In the present study, 18 compounds were selected as marker compounds of Gami-soyosan and a simultaneous analysis was performed for the quality control (QC) of Gami-soyosan by using high-performance liquid chromatography (HPLC)-photodiode array (PDA). The calibration curves of compounds **1**–**18** demonstrated good linear regression with a coefficient of measurement (*r*^2^) of ≥0.9996 (Table 3). By using optimized chromatography, representative chromatograms of the Gami-soyosan sample and standard compounds were obtained (in Figure 2). The concentrations of compounds **1**–**18** were in the range of 0.04 to 8.73 mg/g (Table 4).

Among these compounds of Gami-soyosan, it has been reported that mangiferin (**4**) inhibits osteolysis in ovariectomized rats [31], geniposide (**5**) has an estrogen-like effect by selective ERβ modulation [32], and paeoniflorin (**7**) ameliorates menopausal depression by upregulating serotonin 1A receptor and downregulating serotonin 2A receptor [33]. Furthermore, berberine (**8**) has been reviewed regarding the beneficial effects on menopausal symptoms, such as oxidative stress, dyslipidemia, hyperglycemia, and depression [34]. Like this, mangiferin (**4**), geniposide (**5**), and paeoniflorin (**7**) are considered to be active components against menopausal symptoms in Gami-soyosan, which is prescribed to ameliorate menopausal symptoms. Gami-soyosan is also used to improve similar menopausal symptoms for patients with breast cancer [16,17,18]. We focused on research for the potential utilization of Gami-soyosan by inhibiting the proliferation of ER positive breast cancer cells.

**Figure 3:**
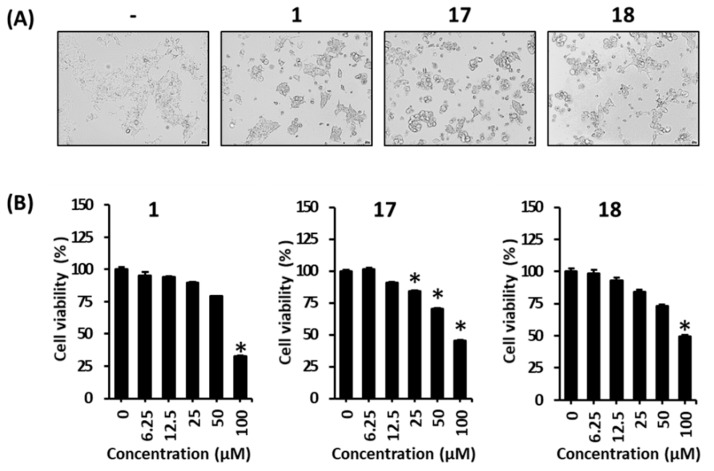
Cytotoxic effects of gallic acid (**1**), decursin (**17**), and decursinol angelate (**18**) in MCF-7 cells exposed to 6.25, 12.5, 25, 50, and 100 μM of each compound for 24 h. (**A**) Viability of MCF-7 cells. (**B**) Morphological changes in MCF-7 cells. The data are presented as the mean ± S.D. and were analyzed using a one-way analysis of variance (ANOVA) and a Dunnett’s multiple comparisons post hoc analysis. * *p* < 0.05 versus untreated cells.

Our preliminary experiments indicated that some Gami-soyosan compounds, such as gallic acid (**1**), decursin (**17**), and decursinol angelate (**18**), suppressed the viability of MCF-7 cells. In this study, the morphological changes induced by these compounds were examined using a phase-contrast inverted microscope. Compared with untreated cells, the treatment with gallic acid (**1**), decursin (**17**), and decursinol angelate (**18**) induced a large number of morphological changes, including membrane blebbing, cell shrinkage, cell condensation, and detachment from the plate (Figure 3A). Cellular morphological changes, such as cell shrinkage, membrane bleb formation, nuclear condensation, and phagocytosis by neighboring cells, are characteristic of apoptotic cell death [35]. Gallic acid (**1**) significantly increased cytotoxicity in a concentration-dependent manner (Figure 3B, IC_50_: 81.3 μM); similar results were obtained for decursin (**17**) and decursinol angelate (**18**) (Figure 3B, IC_50_: 90.8 and 99.3 μM, respectively). These results suggested that treatment with gallic acid (**1**), decursin (**17**), and decursinol angelate (**18**) induces apoptotic cell death.

Gallic acid is an indicator component of Paeoniae Radix (the roots of *Paeonia lactiflora* Pallas) [19]. It promotes the expression of Bax, which induces cancer cell apoptosis and suppresses the expression of apoptosis inhibitory proteins [36]. Through these mechanisms, gallic acid exerts anticancer effects in esophageal, stomach, colon, and uterine cancer cells. In particular, in MCF-7 cells, gallic acid increases p27^kip1^, which inhibits cell cycle regulation and controls MCF-7 cell proliferation through its preventative action on cell division in the G_2_/M phase of the cell cycle [37].

Decursin and decursinol angelate are indicator components of Angelicae Gigantis Radix (roots of *Angelica gigas* Nakai) [19]. They are known to suppress cell division in the G1 phase of the MCF-7 cell cycle and act as anti-cancer agents by inhibiting the mRNA and protein expression of ERα [38]. They also suppress vascular endothelial growth factor (VEGF)-induced phosphorylation of VEGF-2 and prevent angiogenesis, which plays a key role in the growth of cancerous tumors [39].

**Figure 4:**
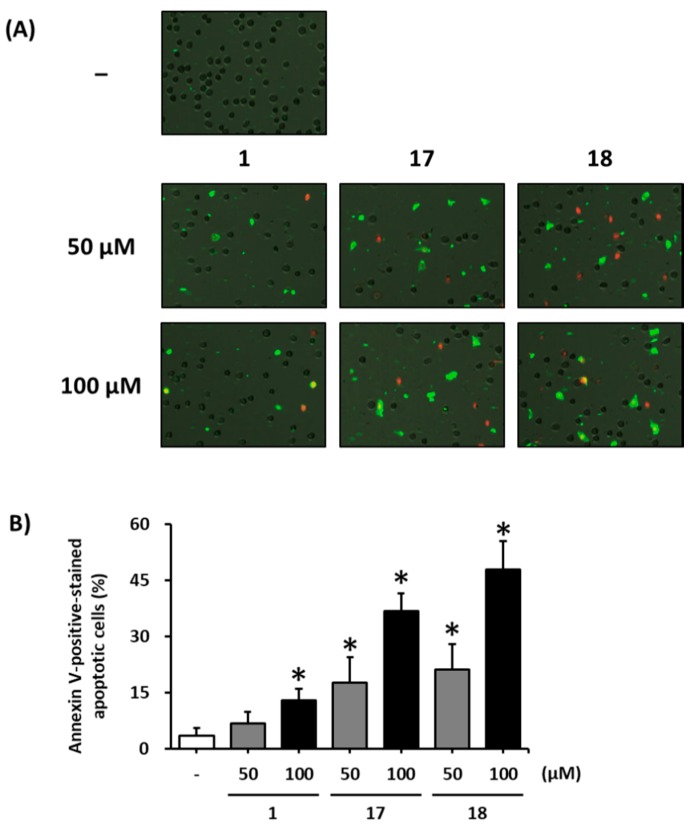
Apoptotic effects of gallic acid (**1**), decursin (**17**), and decursinol angelate (**18**) on MCF-7 cells treated to indicated concentrations for 24 h. The apoptotic cell ratio was analyzed by using Alexa Fluor 488 annexin-V conjugate staining. (**A**) Representative images of detection for apoptotic cells. (**B**) The ratio of apoptotic cells stained positive for annexin V. The data are presented as the mean ± S.D. and were analyzed using a one-way analysis of variance (ANOVA) and a Dunnett’s multiple comparisons post hoc analysis. * *p* < 0.05 versus untreated cells.

To investigate the apoptotic effects of gallic acid (**1**), decursin (**17**), and decursinol angelate (**18**) on MCF-7 cells, the apoptotic cell ratio was analyzed using Alexa Fluor 488 annexin-V conjugate staining. Fluorescent images of cells exposed to these compounds indicated that they induced apoptotic cell death, as shown by the green stain (Figure 4A). The ratio of apoptotic cells significantly increased, from 6.8% to 12.9%, upon treatment with 50 and 100 μM gallic acid (**1**) (Figure 4B). Both decursin (**17**) and decursinol angelate (**18**) strongly increased the percentage of apoptotic cells to a larger extent than gallic acid (**1**) at the same concentration (Figure 4B, 17.7% and 36.9% and 21.2% and 48.0%, respectively).

Apoptosis occurs via extrinsic or intrinsic pathways [40,41]. The extrinsic pathway is regulated by signals from other cells and is initiated by FAS (Fas cell surface death receptor), TNF-α (tumor necrosis factor-alpha), and andligands that penetrate the cell membrane. Subsequently, caspase-8, -3, or -7 are activated and apoptosis occurs. The intrinsic pathway, also called the mitochondrial pathway, is predominantly controlled by the Bcl-2 (B-cell lymphoma 2) family, which comprises anti-apoptotic and pro-apoptotic proteins [42,43,44,45]. Bcl-2 and Bcl-xl, which are representative anti-apoptotic proteins, inhibit the penetration of the mitochondrial membrane and suppress apoptosis while disrupting the action of pro-apoptotic proteins, such as Bax (Bcl-2-associated X protein) and Bak (BCL2-antagonist/killer). However, if these proteins do not function correctly, Bax promotes apoptosis through the induction of caspase-9, -3, and-7 activation. Following the intrinsic and extrinsic pathways, the executioner caspases, caspase-3, -6, and-7, are activated. This in turn causes continuous morphological and biochemical changes in apoptotic cells through the activation of various substances, such as cytokeratin and PARP (Poly (ADP-ribose) polymerase) [45,46,47].

Gallic acid (**1**), decursin (**17**), and decursinol angelate (**18**) exert their action in this pathway and induce the apoptotic death of MCF-7 cells by increasing pro-apoptotic proteins and decreasing anti-apoptotic proteins.

**Figure 5:**
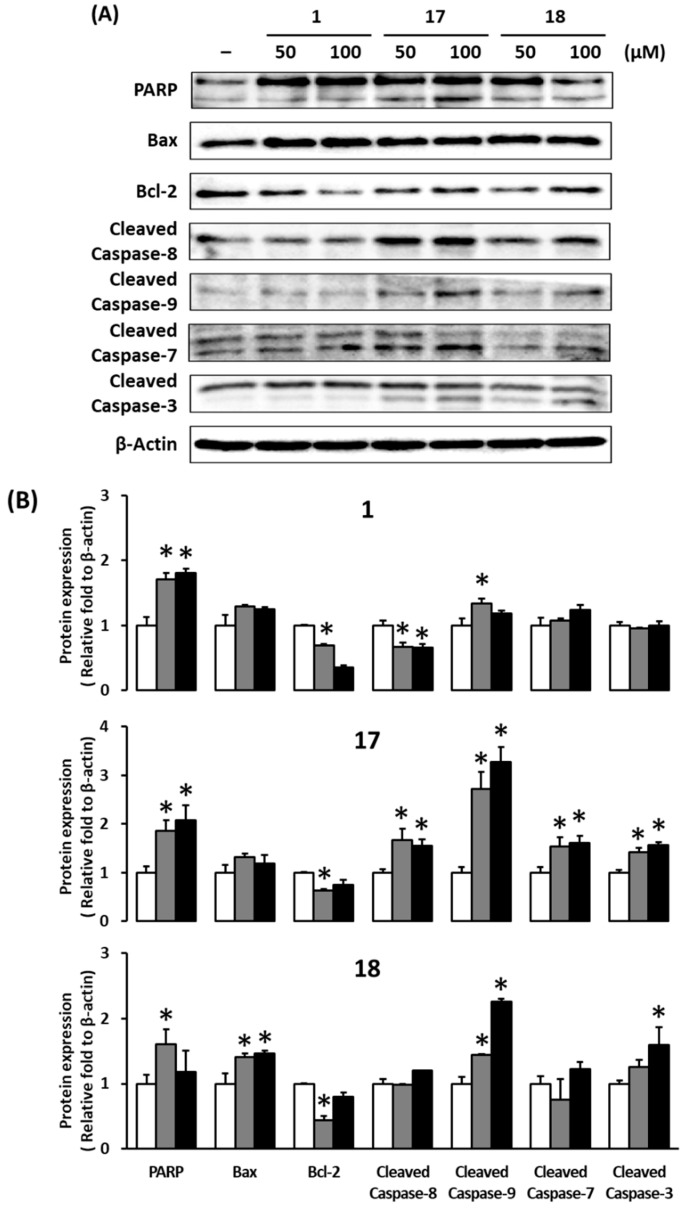
Effects of gallic acid (**1**), decursin (**17**), and decursinol angelate (**18**) on apoptosis in MCF-7 cells exposed to 50 and 100 μM for 24 h, determined by western blotting. (**A**) Protein expression of poly (ADP-ribose) polymerase (PARP), Bcl-2-associated X protein (Bax), B-cell lymphoma 2 (Bcl-2), cleaved caspase-8, -9, -7, -3, and β-actin; (**B**). Graph of relative protein expression. The data are presented as the mean ± S.D. and were analyzed using a one-way analysis of variance (ANOVA) and a Dunnett’s multiple comparisons post hoc analysis. * *p* < 0.05 versus untreated cells.

We performed Western blotting to investigate the apoptotic mechanisms of gallic acid (**1**), decursin (**17**), and decursinol angelate (**18**). In MCF-7 cells, gallic acid (**1**), decursin (**17**), and decursinol angelate (**18**) are known to decrease the expression of Bcl-2 level while increased expression of Bax and cleaved PARP [36,38,48]. In our present study, gallic acid (**1**), decursin (**17**), and decursinol angelate (**18**) significantly increased the expression of cleaved PARP, a general marker of apoptosis (Figure 5A,B). Furthermore, decursinol angelate (**18**) exerted an up-regulation of Bax expression (pro-apoptotic protein) and down-regulation of the Bcl-2 expression (anti-apoptotic protein). In contrast, gallic acid (**1**) and decursin (**17**) down-regulated the expression of Bcl-2 only.

Although the results of Bax were differently expressed, gallic acid (**1**), decursin (**17**), and decursinol angelate (**18**) increased the Bax/Bcl-2 ratio and PARP, respectively. It has been shown that these compounds cause apoptosis in MCF-7 cells via PARP cleavage and the Bax/Bcl-2 ratio increase.

In MDA-MB-231 cells, gallic acid (**1**) enhanced the activity of initiator caspase-8 and -9 MCF-7 cells [49]; however, our study showed that the expression of cleaved caspase-9 was significantly increased in MCF-7. The present study demonstrated that gallic acid (**1**) induced only extrinsic apoptotic pathways in MCF-7 cells.

Treatment with decursin (**17**) also increased the protein expression of cleaved caspase-9 (apoptotic initiator caspase) and -3 (apoptotic effector caspase) in a concentration-dependent manner. In addition, decursin (**17**) significantly increased the expression of cleaved caspase-8 (apoptotic initiator caspase) and -7 (apoptotic effector caspase). These results demonstrated that decursin induced both the intrinsic and extrinsic apoptotic pathways in MCF-7 cells.

In contrast with decursin (**17**), decursinol angelate (**18**) induced the protein expression of cleaved caspase-9 and -3 without changing caspase-8 and -7. Decursin (**17**) and decursinol angelate (**18**) are structural isomers on the side chain [38]. We found that the distinguished structures of the side chain affect the activation of apoptosis related caspases.

In summary, we carried out a simultaneous analysis of 18 compounds of Gami-soyosan by using HPLC-PDA. We demonstrated the apoptotic effects of gallic acid (**1**), decursin (**17**), and decursinol angelate (**18**) on MCF-7 human breast adenocarcinoma cells by using cell viability assays, image-based cytometric assays, and western blotting. Gallic acid (**1**), decursin (**17**), and decursinol angelate (**18**) displayed cytotoxic effects in MCF-7 cells and increased PARP cleavage and the Bax/Bcl-2 protein ratio. Of these compounds, decursin (**17**) and decursinol angelate (**18**) exhibited more potent apoptotic effects in MCF-7 cells than gallic acid (**1**). In particular, they increased the cleavage of caspase-8, -9, -7, and -3. Taking these results together, the components of Gami-soyosan, including gallic acid (**1**), decursin (**17**), and decursinol angelate (**18**), induced the intrinsic or extrinsic apoptotic pathways in ER-positive MCF-7 breast cancer cells.

## 4. Conclusions

We established a method to simultaneously analyze 18 compounds of Gami-soyosan, a prescription used to improve menopausal symptoms. Among the various compounds of Gami-soyosan, gallic acid (**1**), decursin (**17**), and decursinol angelate (**18**) were found to promote apoptosis in MCF-7 cells by increasing pro-apoptotic and decreasing anti-apoptotic protein levels. In conclusion, gallic acid (**1**), decursin (**17**), and decursinol angelate (**18**) may be the Gami-soyosan compounds that inhibit cell proliferation in ER-positive breast cancer. These results suggest that gallic acid, decursin, and decursinol angelate, which are components of Gami-soyosan, may be used to inhibit cell proliferation synergistically in patients with positive breast cancer.

## Figures and Tables

**Table 1:** Composition of Gami-soyosan.

Latin Name	Scientific Name	Amount (g)	Origin
Paeoniae Radix	*Paeonia lactiflora* Pallas	638.3	Uiseong, Korea
Atractylodis Rhizoma Alba	*Atractylodes macrocephala* Koidzumi	638.3	China
Anemarrhenae Rhizoma	*Anemarrhena asphodeloides* Bunge	531.9	Kangjin, Korea
Lycii Radicis Cortex	*Lycium chinense* Miller	531.9	China
Angelicae Gigantis Radix	*Angelica gigas* Nakai	531.9	Bonghwa, Korea
Poria Sclerotium	*Poria cocos* Wolf	425.5	Pyeongchang, Korea
Liriope Tuber	*Liriope platyphylla* Wang et Tang	425.5	Miryang, Korea
Rehmanniae Radix Crudus	*Rehmannia glutinosa* Liboschitz var. *purpurea* Makino	425.5	Gunwi, Korea
Gardeniae Fructus	*Gardenia jasminoides* Ellis	266.0	Gurye, Korea
Phellodendri Cortex	*Phellodendron amurense* Ruprecht	266.0	China
Platycodi Radix	*Platycodon grandiflorum* A. De Candolle	159.6	Muju, Korea
Glycyrrhizae Radix et Rhizoma	*Glycyrrhiza uralensis* Fischer	159.6	China
Total		5000.0	

**Table 2:** Chromatographic parameters for high-performance liquid chromatography (HPLC) analysis of 18 marker components in Gami-soyosan.

Chromatographic Parameter			
Column	SunFire C_18_ analytical column (250 × 4.6 mm, 5 μm)		
Detector	PDA (220, 230, 240, 254, 270, 275, 325, 330, 335, and 340 nm)		
Flow rate (mL/min)	1.0		
Injection volume (μL)	10.0		
Column temperature (°C)	40		
Mobile phase	A: 0.1% Formic acid in distilled water B: 0.1% Formic acid in acetonitrile		
Gradient elution	**Time (min)**	**A (%)**	**B (%)**
	0	95	5
	30	40	60
	40	0	100
	45	0	100
	50	95	5

**Table 3:** Detection wavelength, range, regression equation, and coefficient of determination (*r*^2^) of compounds **1**–**18**.

Compound	Detection Wavelength (nm)	Linear Range (μg/mL)	Regression Equation ^a^	*r* ^2^
**1**	270	0.63−40.00	*y* = 38259.49*x* – 10572.16	0.9999
**2**	254	0.63−40.00	*y* = 32295.16*x* – 2577.19	1.0000
**3**	325	0.63−40.00	*y* = 41318.61*x* – 24432.53	0.9996
**4**	254	0.63−40.00	*y* = 50951.35*x* – 13475.92	0.9999
**5**	240	0.63−40.00	*y* = 16710.90*x* – 141.25	1.0000
**6**	230	0.63−40.00	*y* = 10804.67*x* – 2134.28	0.9999
**7**	230	0.63−40.00	*y* = 10839.56*x* + 774.96	0.9997
**8**	340	1.56−100.00	*y* = 60688.22*x* – 23759.65	1.0000
**9**	275	0.63−40.00	*y* = 15209.48*x* – 2448.89	1.0000
**10**	275	0.63−40.00	*y* = 17437.96*x* – 2592.24	1.0000
**11**	335	1.56−100.00	*y* = 32877.31*x* – 14561.30	1.0000
**12**	230	0.31−20.00	*y* = 35372.59*x* – 5310.55	0.9999
**13**	275	0.31−20.00	*y* = 33887.88*x* – 3082.11	1.0000
**14**	230	0.31−20.00	*y* = 38088.40*x* – 3804.89	0.9999
**15**	254	0.63−40.00	*y* = 8282.39*x* – 1435.76	1.0000
**16**	220	0.31−20.00	*y* = 59991.32*x* – 1482.57	0.9999
**17**	330	0.63−40.00	*y* = 37380.95*x* – 4157.31	1.0000
**18**	330	0.63−40.00	*y* = 257615.48*x* – 2550.42	1.0000

^a^ y and x mean peak area (mAU) and concentration (μg/mL) of compounds, respectively.

**Table 4:** Concentration of compounds **1**–**18** in Gami-soyosan (*n* = 3).

Compound	Mean (mg/g)	SD (×10^–1^)	RSD (%)
**1**	0.78	0.86	1.10
**2**	0.50	0.29	0.58
**3**	0.88	0.28	0.32
**4**	2.24	1.39	0.62
**5**	8.57	13.75	1.60
**6**	0.60	0.09	0.14
**7**	8.73	11.83	1.35
**8**	2.75	1.70	0.62
**9**	1.02	0.78	0.77
**10**	1.18	1.82	1.55
**11**	1.56	0.57	0.36
**12**	0.61	0.46	0.75
**13**	0.12	0.27	2.25
**14**	0.14	0.13	0.96
**15**	0.67	0.49	0.73
**16**	0.04	0.06	1.29
**17**	0.56	0.42	0.76
**18**	0.05	0.02	0.47
